# Oxygen as a Therapeutic Agent

**Published:** 1886-11

**Authors:** C. E. Ehinger


ARTICLE III.

### OXYGEN AS A THERAPEUTIC AGENT.

BY C. E. EHINGER, M. D.

### CLINICAL CASES.

CASE 6.— Mrs. H., aet. 33, widow, came to me early in January upon the recommendation of some former patient. States that she had been unable to perform her household duties for about three months, during which time she had been under the treatment of several physicians without any improvement. Her most urgent complaints at the time were insomnia and migratory rheumatism, to which she adds the following list of secondary troubles: anorexia, flatulent dyspepsia, obstinate constipation, and dysmenorrhœa. The simplest food in small quantities caused much distress from distention of the stomach and abdomen and sour eructations. Bowels rarely moved without the aid of laxatives or enema. Rheumatic pains very annoying at all times but much aggravated every morning. Was always awakened by pain which at one time was in the shoulder, then the hip, elbow or ankle.

She was put upon what I term the standard mixture, consisting of oxygen, two volumes; nitrous oxide; two volumes; air, four volumes; commencing with four inhalations daily which were increased gradually until eight inhalations were taken at a treatment. The very first treatment was productive of the happiest results, patient announcing with much satisfaction the next day that she had enjoyed the best night's sleep she had had for months. During the first week little change was noticed in the gastric and enteric symptoms, although she thought she had more desire for food, but still felt distressing after eating. At the end of the second week noticed marked improvement in the appetite, less pyrosis, bowels moved without enema or laxative, though not without some difficulty. Still said she was much troubled with burning in the epigastric region. These symptoms all improved gradually, and a little before the expiration of the month she discontinued the treatment, declaring herself well, which appearances certainly seemed to indicate as she had gained much in flesh and strength.

CASE 7.—Mr. L., aet. 62. Has been a sufferer from asthma and bronchial catarrh for ten or twelve years. The first approach of cold weather has always been a signal for the onset of severe asthmatic symptoms, which not only rendered business burdensome but at times an impossibility. Often paroxysms so severe that he was obliged to spend the night in the easy chair owing to the distress occasioned by a recumbent posture. He began the use of oxygen in January while suffering from a severe attack. He was put upon the modified mixture, two small treatments daily. Pure oxygen was given during the severe paroxysm with the effect of removing the most distressing symptoms at once. Dyspnœa and cough improved from the first, so that at the end of three weeks but one small treatment—of four inhalations—daily, was given. This was continued for a month longer. After the first week there was no return of the distressing symptoms and a decided improvement of the bronchial trouble was manifest. This gentleman remarked incidentally that his bowels had not acted so regularly for years as since commencing the oxygen treatment.

CASE 8.—N. C., aet., 30, colored coachman. Was under treatment for tubercular phthisis at the time I put in my oxygen outfit. As soon as arrangements were completed, he was placed upon the mixture. Condition at this time was as follows: He was confined to his bed, much emaciated, unable to take even liquid nourishment without causing pain in stomach, and greatly aggravating a troublesome diarrhœa. Suffering from profuse night-sweats, bad cough with profuse blood-streaked expectorations, temperature ranging between 100° and 104°. Physical examination.—Numerous moist rales heard at the apex, with signs of two small cavities. Bronco-vesicular breathing in the mammary region. Entire absence of vesicular sounds in subscapular and infra-axillary region. Oxygen was sent to this patient in a rubber gas-bag. The usual proportion was administered at first, which was changed after four or five days, by increasing the proportion of oxygen one volume. The rapidity with which this man gained was almost beyond comprehension. The night-sweats were soon checked, cough and diarrhœa diminished rapidly, temperature declined so that the evening rise was not over 101°. At the end of the first week he put his clothes on and walked about the house, and by the middle of second week surprised me by walking to my office, a distance of three quarters of a mile. In a few days after this he came regularly to my office for treatment. At the expiration of three weeks he wanted to go to work, but to this I would not give my consent; he, however, did some light work about the house. In a month he had gained fifteen pounds. Coughed but little, slept well and had an enormous appetite. But alas! here the good report ends, for about this time an incident occurred which changed the tide of events, and blighted the hopes I had begun to cherish of reporting a case of advanced phthisis arrested by the oxygen treatment. The domestic misunderstanding which resulted so disastrously to my patient and indefinitely postponed the hope I had cherished, was something as follows: One cold night in January the patient under consideration bethought himself that a fire in his sleeping apartment would add very materially to his comfort and well being, but this announcement was greeted by his aged mother as an unwarranted extravagance, for the very thought of which he was severely admonished, and sarcastically reminded that the summer kitchen was warm enough, a night like that, and withal good enough for a worthless being who would harbor such high-flown notions. The upshot of the matter was that our patient repaired to the summer kitchen, built a fire in a rickety stove there, and turned in for the night upon an ironing board. Toward morning, the fire having gone out, he awakened with a chill, and the next day had a slight hemorrhage. From this time on the oxygen proved unavailing, and in a month's time the patient ceased to be.

I realize that some of my hearers will smile at the inglorious termination of the case and mentally scoff at the idea of the occurrence related being even indirectly responsible for his death. Be that as it may, the writer will ever adhere to the conviction that his promising patient was sacrificed a martyr to rigid economy and willful cussedness.

CASE 9.—Mrs. W., aet. 37, married, came to me for the relief of an unusually obstinate cough, of between four and five years standing. Has been under the treatment of a most excellent physician, who had studied her case carefully and done everything in his power for her relief, but failed to give more than temporary benefit. Cough was paroxysmal, severe seizures occurring at night which continue uninterruptedly for half to three-quarters of an hour, and are often frequently repeated, thus greatly interfering with and sometimes abolishing sleep. Paroxysms frequently occur while eating which compel her to retire from the table, and sometimes result in her losing what food she had taken. At times complains of circumscribed soreness in upper part of right lung. Repeated examination of lungs have been made by her physician but always with negative results, nothing of importance being discovered. It was thought that the presence of an hypertrophied tonsil and slight follicular pharyngitis might be the cause, and they were energetically attacked and much relieved, but with no effect upon the cough. The question of "trigeminal cough," "liver cough," "ear cough," "stomach cough," "ovarian," "uterine," and various other coughs were successively canvassed, and duly discarded as untenable. Some good ground existed for considering the cough of gastric origin, since the patient was a great sufferer from atonic dyspepsia, accompanied by an usually torpid state of the bowels, no evacuation occurring sometimes for a week unless enemas were resorted to. All the ordinary and some oxtraordinary remedies were tried unavailingly, much to the disgust of both patient and doctor. For some time before commencing the use of oxygen the patient had taken no treatment whatever. Hearing of a similar case being cured by oxygen she consulted me and finally decided to give the oxygen a trial.

I feel some hesitation in giving the exact results obtained, lest either my veracity or sanity be questioned; however, as "truth is mighty," I will risk the consequences and state the unvarnished facts. The first treatment was followed by a night of perfect rest uninterrupted by a single seizure of coughing, something unknown for over a year. I do not mean to convey the impression that the cough ceased forever, but it did continue to improve steadily. The appetite returned, gastric symptoms disappeared and the constipation was much relieved, though in this case, not cured. The treatment was taken for a month, patient deeming further treatment unnecessary. I will briefly relate one more case before bringing my already too long article to a close.

CASE 10.—Mr. C., æt. 33, married, an engraver. One year ago last winter, he suffered a severe attack of double pneumonia; perfect resolution did not result, indurations remaining in both lungs; he also continued to have a troublesome cough and dyspnœa. Lung capacity on commencing treatment was 154 cubic inches. In two weeks it had increased to 175 cu. in., and both cough and dyspnœa were much diminished. Patient took the treatment a month, at the end of this time he thought his lungs as good as ever, chest capacity in the mean time having increased to 210 cubic inches.—*Medical Current.*

---

